# PIVKA-II: A biomarker for diagnosing and monitoring patients with pancreatic adenocarcinoma

**DOI:** 10.1371/journal.pone.0251656

**Published:** 2021-05-20

**Authors:** Sara Tartaglione, Patrizia Mancini, Valentina Viggiani, Piero Chirletti, Antonio Angeloni, Emanuela Anastasi

**Affiliations:** 1 Department of Experimental Medicine, "Sapienza" University of Rome, Policlinico Umberto I, Rome, Italy; 2 Department of Molecular Medicine, "Sapienza" University of Rome, Policlinico Umberto I, Rome, Italy; 3 Department of Surgical Sciences, "Sapienza" University of Rome, Policlinico Umberto I Policlinico Umberto I, Rome, Italy; Centre de Recherche en Cancerologie de Lyon, FRANCE

## Abstract

**Background:**

Pancreatic adenocarcinoma (PDAC) is an incurable cancer without adequate tumor markers. Our previous study has showed a better diagnostic performance of Protein Induced by Vitamin K Absence II (PIVKA-II) compared to currently used PDAC biomarkers. To corroborate our previous data with a larger sample size and to assess a possible role of PIVKA-II in predicting surgical success. Additionally, to further evaluate the hypothesis of a direct PIVKA-II production by PDAC cells, we examined PIVKA-II tissue expression in a case of PDAC using immunofluorescence.

**Methods:**

We enrolled 76 newly diagnosed PDAC patients and selected 11 patients to determine PIVKA-II levels also after surgical resection. An immunofluorescence (IF) study of PIVKA-II tissue expression was carried out in one of them. PIVKA-II serum values were measured by chemiluminescent enzyme immunoassay method (CLEIA) on LUMIPULSE G1200 (Fujirebio-Europe, Belgium).

**Results:**

PIVKA-II serum levels were above the cut-off at baseline in 71 patients (94%) with a median value of 464 mAU/Ml (range 27–40783 mAU/mL); the sensitivity and specificity were 78.67% and 90.67% respectively. Patients with pre-operative PIVKA-II positivity showed a significant decrease (P < 0.015) of median PIVKA-II serum concentrations after surgery: 820 (91–40783) mAU/mL at diagnosis vs 123 (31–4666) mAU/mL post-operatively. IF assay on PDAC sections demonstrated PIVKA-II expression in cancer cells.

**Conclusion:**

These data are the first showing a decreased PIVKA-II serum levels after surgery in PDAC patients and reporting PIVKA-II expression in PDAC tissue. Further studies are needed to confirm these findings and to determine PIVKA-II usefulness in diagnosing and monitoring PDAC patients.

## Introduction

Pancreatic cancer is a lethal malignancy representing the seventh leading cause of global cancer deaths in industrialized countries. Pancreatic adenocarcinoma (PDAC) arising in exocrine glands of the pancreas, is the most common (85% of cases) type of pancreatic cancer [1]. Only 5–7% of patients survive 5 years after diagnosis and surgical excision is the only potential curative treatment with a 5 years survival rate of 20% [2]. Unfortunately, due to lack of specific symptoms 80% of patients are diagnosed at an advanced or metastatic stage of disease when their tumors are not surgically resectable [3]. Additionally, the risk for systemic recurrence can involve ~ 80% of resected patients [4]. There are a number of reasons related with the poor prognosis associated with PDAC but one of the main causes is the difficult in early identification [5]. The opportunity to detect pancreatic cancer while it remains curable is crucial for better prognosis, and besides standard diagnostic approaches such as imaging techniques, easily accessible biomarkers might be of critical importance for a timely diagnosis. Because of the insufficient individual sensitivity or specificity of already established PDAC biomarkers, there is a constant ongoing effort to identify additional ones [6]. In a recent preliminary study investigating a novel serum biomarker for PDAC, we evaluated Prothrombin induced by vitamin K absence-II (PIVKA-II) values in a cohort of PDAC Italian patients [7]. PIVKA-II is an abnormal prothrombin containing some glutamic acid (Glu) residues. When prothrombin is generated by liver cells under conditions of reduced vitamin K or in the presence of a vitamin K antagonist, the Glu residue at the N-terminal of the prothrombin precursor is not completely converted to γcarboxyglutamic acid (Gla) by γ-carboxylase [8]. Until the 1980’s, the resulting protein, PIVKA-II, was mainly used as an indicator of blood coagulability but since Liebman et al. in 1984 reported detection of a high rate of serum PIVKA-II in hepatocellular carcinoma (HCC) patients, this biomarker has been widely used for this neoplasm and it presently represents an important tool in the diagnosis and prognosis [9–11]. Anyway it has recently been reported (mainly from Japan) the presence of elevated PIVKA-II values in different kinds of in other gastrointestinal neoplasms such as gastric cancer, colon cancer and PDAC [7]. The mechanism of PIVKA-II production in non HCC tumors is currently unknown. Hepatoid differentiation of tumors has been speculated as one of the mechanisms of PIVKA-II production [12]. However it has been showed that 10 of 23 reported cases of PIVKA-II producing tumors (43.4%) did not have hepatoid differentiation. It has been demonstrated that PIVKA-II producing tumors show a high rate of liver metastasis and poor prognosis [13]. In our pilot study we reported for the first time that PIVKA-II is significantly higher in PDAC than in benign pancreatic diseases and that it shows a rather good diagnostic performance compared to commonly used tumor markers Carbohydrate Antigen 19.9 (CA 19.9), Carcino-Embryonic Antigen (CEA) and CA242 [7]. Since our results were promising, the current study aimed to corroborate our previous data with a larger sample size and to assess a possible role of PIVKA-II in predicting surgical success. Additionally, to further evaluate the hypothesis of a direct PIVKA-II production by PDAC cells, we examined PIVKA-II tissue expression in a case of PDAC using immunofluorescence (IF).

## Materials and methods

### Patient demographics

From January 2018 to December 2019, a random sample of patients has been selected for the study. Of 87 possible patients, 76 were chosen and gave their informed consent to participate in the study. The selection method was that every patient diagnosed with PDAC at the Oncologic Unit A, of Policlinico Umberto I, Rome, Italy, from monday to friday. Patients were excluded if their alcohol assumption was higher than a single serving day, if they showed evidence of an active hepatopathy. if they took anti Vitamin K antagonists or if they had any coagulopathy. We excluded 11 subjects so the final patient cohort was made up of 76 sera, while 55 sera from healthy, cancer-free blood donors served as control samples. The PDAC patients and healthy control cohorts had similar demographics in terms of ratio of men to women, with the majority of all subjects being Caucasian. Table 1 shows patients and controls characteristics. PDAC patients met the following eligible criteria: adult age (≥ 18 years), the first occurrence of neoplastic pathology, no prior treatment with neoadjuvant therapy, absence of diabetes, no serious physical disabilities. The study protocol was approved by Policlinico Umberto I Review Board and all partecipants in the study, patients and volunteers, signed a written informed consent. At enrolment (T0), medical history was collected for each subject, and peripheral blood samples were drawn and immediately sent to the laboratory of Tumour Markers of Policlinico Umberto I, Rome, Italy. When histopathological and radiological examination confirmed the presence of PDAC each serum sample was analyzed for PIVKA-II. We selected 11 patients for assessing PIVKA-II levels after surgical resection (T1) of neoplasm and stratified them into 2 groups by tumor marker level as follows: positive PIVKA-II group (n. 7) and negative PIVKA-II group (n. 4). In order to avoid complicating factors, we chose only cases without any preoperative treatment. Demographics and clinical characteristics of PDAC patients after surgery are presented in Table 2. All 11 patients were subjected to postoperative histopathological diagnosis, which confirmed the presence of PDAC and Tumour, Node, Metastasis (TNM) classification and American Joint Committee on Cancer (AJCC) classification was used for the clinicopathological parameters of their tumor. Intraoperative tissue specimens of a patient among the 11 resected ones was chosen for immunohistochemical and IF studies. The study protocol was approved by Policlinico Umberto I Review Board and was performed in accordance to Helsinki Declarations. All partecipants in the study, patients and volunteers, signed a written informed consent.

**Table 1:** Demographics and clinical characteristics of PDAC study cohort and controls.

Parameter	PDAC patients	Controls
Number of cases	76	55
Age (years, median and range)	73 (40–92)	71 (38–65)
Sex male-female (%)	42–58	24–31
No smoker-smoker (%)	58–42	79–21
Ethnicity (%) caucasian-others	97–3	51–4
Hypertension	26	5
Respiratory diseases	8	1
Dyslipidemia	18	5
CA 19.9 (median and range)	260 (33–290)	39 (9–51)
PIVKA II (median and range)	464 (27–40783)	29 (13–49)
Tumor grade		
I	21	-
II	44	-
III	8	-
IV	3	-

**Table 2:** Demographics and clinical characteristics of PDAC patients after surgery.

Parameter	PDAC patients showing PIVKA reduction after surgery	PDAC patients not showing PIVKA reduction after surgery
Number of cases	7	4
Age (years, range)	73 (40–92)	71 (38–65)
Sex male-female	2–5	3–1
No smoker-smoker	2–5	1–3
Ethnicity (%) caucasian-others	100	100
Hypertension	2	0
Respiratory diseases	0	0
Dyslipidemia	3	0
CA 19.9 (median and range)	270 (102–290)	301 (186–290)
PIVKA II (median and range)	580 (474–40783)	516 (283–39571)
Tumor grade		
III	5	3
IV	2	1

#### Blood sampling

Blood collection was performed following a standard protocol. Peripheral blood samples were obtained by venous puncture, collected in a red top Vacutainer (Becton, Dickinson and Company, Plymouth, UK) clotted 60–90 minutes and centrifuged for 10 minutes at 1300xg. The serum fractions were aliquoted in 1.5 mL Eppendorf tubes (Eppendorf srl, Milano, Italy) and stored at– 80°C until analysis.

### Methods

#### Serum PIVKA-II determination

PIVKA-II serum values were measured on a Lumipulse G1200 (Fujirebio-Europe, Gent, Belgium), using the LUMIPULSE G PIVKA-II kit (Fujirebio, Tokyo, Japan) a full automated instrument based on elettrochemiluminescence (CLEIA) technology. The intra and inter-assay CV were respectively < 2.4% and < 10% while the detection range was between 5–75,000 mAU/mL. We considered the clinical cut off of 48 mAU/mL, as we determined within our laboratory in our previous pilot study [7,14]. All assays were performed in duplicate.

#### Collection of post operatory tissue and IF protocol

Immunohistochemical and IF studies were performed on one case of PDAC. Immunohistochemistry was carried out on formalin-fixed paraffin-embedded 5 μm thick tissue sections stained with conventional Hematoxylin and eosin (H&E) staining protocol [15]. The tissue sections were analysed by an Olympus BX52 microscope (Olympus Italia, Milano, Italy); imagines were acquired and processed by the IAS 2000 software (Olympus). Slides were immunostained in the same batch, to prevent incubation variability and to ensure identical condition for comparison. IF was performed on Frozen Optimal cutting temperature compound (OCT) blocks of PDAC cut using a cryostat into 5-μm thick sections. We used an unconjugated primary mouse monoclonal antibody directed to PIVKA II (PIVKA II antibody orb 422529, Biorbyt) followed by a fluorophoreconjugated secondary polyclonal rabbit anti-mouse antibody (F0261, Dako) at 1:40 diluition. Serial dilutions of primary antibodies were tested till finding the optimal one, 1: 100. Nuclei were subsequently counterstained with 4′, 6-diamidino-2-phenylindole (DAPI) (Invitrogen). IF signal of PIVKA-II was analyzed by recording stained images using an Axio Observer Z1 inverted microscope, equipped with an ApoTome.2 System (Carl Zeiss Inc., Ober Kochen, Germany). The ApoTome system provides an optical section of fluorescent samples, calculated from three images with different grid positions without time lag. Digital images were acquired with the AxioCam MRm high resolution digital camera (Zeiss) and processed with the AxioVision 4.8.2 software (Zeiss) [16]. Slides with absence of the primary antibody were also included as negative controls. In order to make a comparison between the histological samples and those treated for IF, we used the samples that came from the same experimental preparation.

### Statistical analysis

The results are expressed as median and range. To evaluate the discrimination ability of the tested biomarker, area under the receiver operating characteristic curve (ROC AUC) was calculated. For AUC we estimated the 95% confidence interval (95% CI). The values P < 0.05 were considered statistically significant. Relationships between PIVKA-II positivity and the clinicopathological parameters were analyzed with the Chi-2 and Mann–Whitney U-tests. Statistical analysis was performed using StatsDirect 3.0.187 statistical software (StatsDirect software, Cheshire, England)

## Results and discussion

### PIVKA-II serum levels are increased at time of PDAC diagnosis (T0)

Patients’ characteristics at baseline are presented in Table 1. We observed significantly elevated levels of circulating PIVKA-II in PDAC patients compared to control samples: the median baseline PIVKA-II serum levels were 464 mAU/mL (range 27–40783 mAU/mL) in PDAC patients while healthy subjects had median PIVKA-II serum levels of 33 mAU/mL (range 13–39 mAU/mL) (P < 0.02). The evaluation of PIVKA-II showed a positivity of this biomarker in 71 PDAC patients (94%) while only one of the controls showed PIVKA-II serum levels above the selected cut- off. In ROC curve analysis, PIVKA-II showed an AUC value of 0.9 for the differentiation between PDAC patients and healthy control samples. At the ideal cut-off value of 48 mAU/mL that we established using the Youden index method, PIVKA-II showed a sensitivity of 78.67% with a specificity of 90.67% for the diagnosis of PDAC.

### PIVKA-II serum levels after surgery (T1) decrease in patients with pre- operative PIVKA-II positivity

Following PDAC resection, PIVKA-II serum levels significantly decreased from baseline to T0–T1 in patients with increased PIVKA-II at time of diagnosis (P < 0.015): from 820 (91–40783) mAU/mL to 123 (31–4666) mAU/mL. On contrary, in patients with PIVKA-II in normal range before surgical resection, we didn’t observe a significative decrease of PIVKA-II serum levels from baseline to T1: from 47 (36–48) mAU/mL to 44 (28–45) mAU/mL.

### PIVKA-II is expressed in PDAC tissue

**Fig 1:**
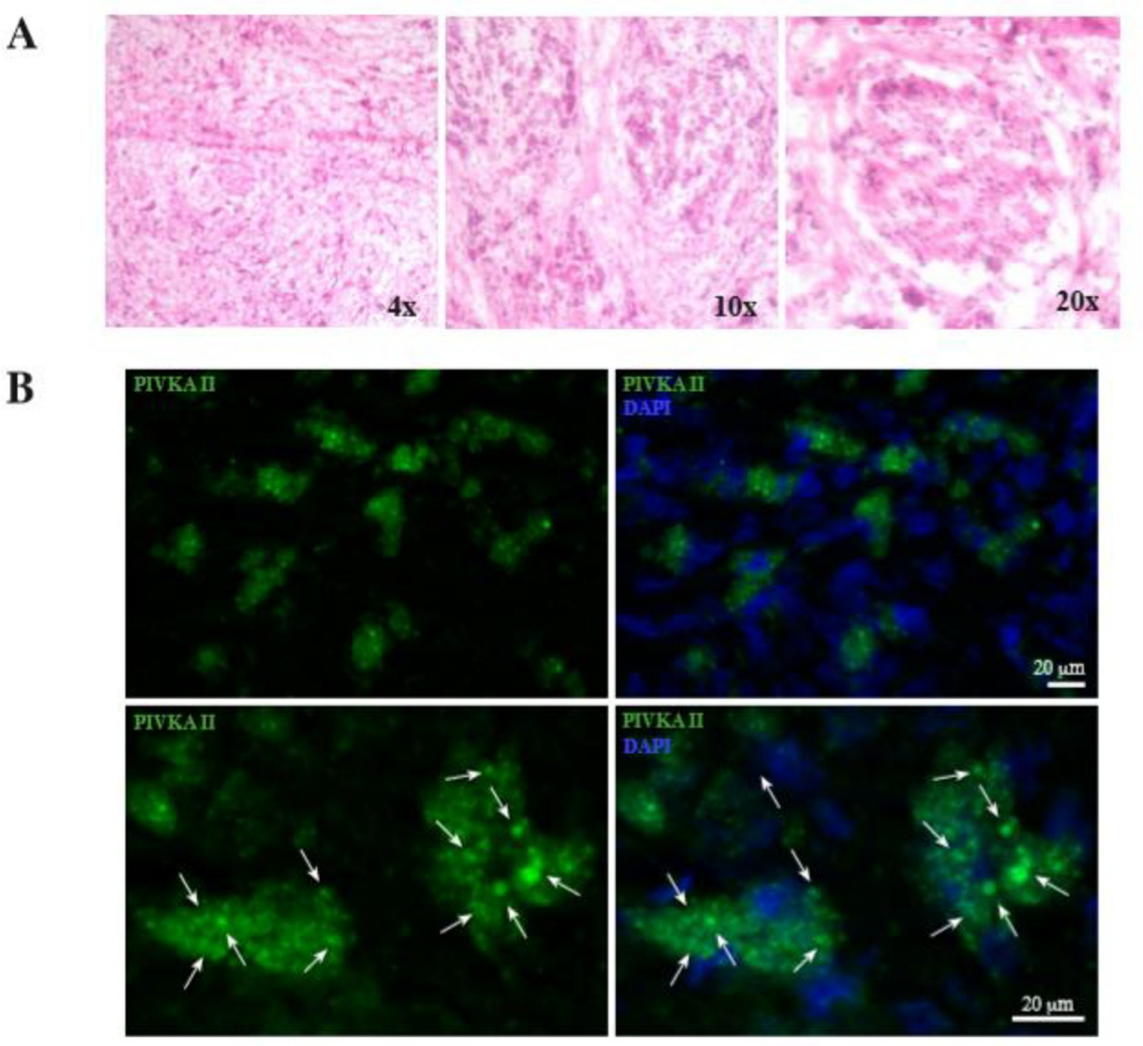
PIVKA II espression in PDAC tissue slides. A) H&E staining of PDAC sections; the tissue is intact and composed entirely of tumor cells, pleomorphic and with altered cytoplasmic nucleus ratio. B) IF analysis of the PIVKA II of PDAC tissue. Several cells are marked, and the signal appears cytoplasmatic and dotted, mainly distributed in the perinuclear area. DAPI was used to highlight cell nucleus. Arrowheads indicate cytoplasmic dots. Bar: 20 μm. H&E = Hematoxylin and eosin PDAC = Pancreatic adenocarcinoma IF = immunofluorescence DAPI = 4′,6-diamidino-2-phenylindole.

To analyze the tissue morphology, we stained PDAC tissue slides with hematoxylin eosin. Fig 1A shows that the tissue appeared intact and composed entirely of tumor cells, pleomorphic and with altered cytoplasmic nucleus ratio. Furthermore, to evaluate the expression of PIVKA-II on PDAC tissue, we performed IF assay on PDAC sections, which came from the same experimental preparation of the histologic samples. Results obtained demonstrate that several PDAC cells expressed PIVKA-II protein, as revealed by the staining observed in Fig 1B. Marked cells showed a vesicular cytosolic pattern of the fluorescent signal, with vesicles of different sizes, mainly arranged in the perinuclear area, suggesting an accumulation of the protein within the cytoplasmic dots (Fig 1B, arrowheads).

In the present research we showed that PIVKA-II is an effective biomarker of PDAC diagnosis, confirming the data of our previous pilot study [7]. Moreover, we found that serum PIVKA-II levels are reduced in PDAC patients after surgical treatment. We also demonstrated PIVKA-II tissue positivity in one case of PDAC. In a recent paper we reported for the first time that serum PIVKA-II is increased in a cohort of PDAC Italian patients: to corroborate our data we decided to enlarge the sample size in the current study and we obtained encouraging results since 71 patients (94%) had PIVKA-II above the cut off. This biomarker showed a sensitivity of 78.67% and a specificity of 90.67% for the diagnosis of PDAC, a dismal neoplasm which has an urgent need of reliable diagnostic tools. Indeed, among PDAC patients only 10–20% represent potential candidates for a curatively intended surgical resection, since the majority of patients is diagnosed with an advanced, non-resectable tumor stage also due to the vagueness of initial symptoms [17]. Furthermore, the recurrence rate after resection is ~ 80% after five years [18]. The available diagnostic tests are non-specific and consequently may miss patients with early-stage disease or not detect a relapse: for this reason an abundance of research in recent years has focused on identifying biomarkers for PDAC diagnosis, prognosis and to evaluate surgical success [19]. Regrettably, today the diagnostic tools to preoperatively characterize the aggressiveness of the individual patient’s PDAC are limited because no satisfactory biomarkers are currently available: the only Food and Drug Administration (FDA) approved for monitoring PDAC patients is CA 19.9 which has relatively good sensitivity but poor specificity [20,21]. Accordingly, our aim was determining if PIVKA-II could represent an effective help on patients who underwent curative resection to support CA 19.9 and imaging for assessing surgical success in PDAC. It has been demonstrated that measuring PIVKA-II serum levels before and after HCC treatment is clinically useful in monitoring of treatment outcomes and prognosis and in predicting recurrence [22]. To our knowledge, this is the first research demonstrating that PIVKA-II serum level could have a prognostic value when used as a biomarker for monitoring surgical success in patients with PDAC. Our results showed reduced circulating PIVKA-II levels after curative surgery in PDAC patients: taking serologic response as indicative of reduced tumor load is based on the premise that baseline high PIVKA-II levels are due to direct PDAC cells production. So, in light of these considerations and in order to validate PIVKA-II diagnostic role and to prove that PIVKA-II production could be a prognostic factor of PDAC, we assessed the expression of tissue PIVKA-II. Since our study is the first exploring tissue PIVKA-II in PDAC, we elaborated an experimental protocol to confirm that this biomarker is expressed specifically in neoplastic cells and that it isn’t a hepatic ectopic production, a paraneoplastic effect or that it is caused by a vitamin K deficit [23]. Tissue PIVKA-II positivity showed in one of our studied resected case is in line with the serological findings and strengthen our hypothesis. The presence of PIVKA-II in PDAC tissue, therefore its production by PDAC cells, opens the way for the possibility of using this protein as a marker in the pathogenetic, diagnostic or prognostic field, for its presence not only in the serum, but also at tissue level. In this context PIVKA-II tissue expression could be used to better understand PDAC biology as well as to eventually study the fate and the role of this tumor marker during the initiation, progression, and metastasis of PDAC. Determining if there is a clinicopathologic significance of PIVKA-II serum and tissue expression in PDAC this study would allow to clarify PIVKA-II role in early diagnosis or determining prognosis of this type of cancer. In combination with radiological assessment, PIVKA-II may be useful to monitor surgical outcomes in this cancer patients and the information derived from serum and tissue PIVKA-II can represent an accurate tool for the oncologists in PDAC cancer management. In conclusion, our study shows that PIVKA-II is a robust diagnostic biomarker, and a marker of surgical success in a cohort of Italian PDAC patients with resectable disease. We also showed for the first time PIVKA-II tissue expression in this type of neoplasm. We are aware that the present study has some potential drawbacks: this is a single centre study with a small sample size, expecially considering postoperative sampling. Additionally, our study was not designed to report long term outcomes of these patients, or the association between normalization of PIVKA II and recurrence free and overall survival. Despite these limitations, we think that our findings deserve specific prospective validation in population with higher number of patients to confirm the diagnostic and potential prognostic value of PIVKA-II in patients with PDAC. If our encouraging data will be confirmed in further large-scale and multi center studies, we will have an additional prognostic tool suitable for clinical routine evaluation, low-cost, and with high reproducibility [24].

## Conclusions

The data of this investigation confirm that PIVKA II is a serum biomarker increased in PDAC patients, as it has been recently reported [7]. Moreover, our results are the first showing a decreased PIVKA-II serum levels after surgery in PDAC patients and reporting PIVKA-II expression in PDAC tissue. PIVKA-II may be useful to monitor surgical outcomes in this cancer patients and the information derived from serum and tissue PIVKA-II can represent an accurate tool for the oncologists in PDAC cancer management. Further prospective studies on larger cohorts of patients are needed to confirm the findings of the present investigation.
